# Exercise effects on symptoms of depression and anxiety vary by patient, clinical, and intervention characteristics in cancer survivors: Results from pooled analyses of individual participant data of 26 RCTs

**DOI:** 10.1007/s00520-025-09646-9

**Published:** 2025-07-01

**Authors:** Marlou-Floor Kenkhuis, Meike Doorenbos, Isa H. Mast, Neil K. Aaronson, Marc van Beurden, Martin Bohus, Kerry S. Courneya, Amanda J. Daley, Daniel A. Galvão, Martine M. Goedendorp, Wim H. van Harten, Sandi C. Hayes, Anouk E. Hiensch, Melinda L. Irwin, Marie José Kersten, Hans Knoop, Anne M. May, Alex McConnachie, Willem van Mechelen, Nanette Mutrie, Robert U. Newton, Frans Nollet, Hester S. Oldenburg, Martina E. Schmidt, Kathryn H. Schmitz, Karl-Heinz Schulz, Gabe S. Sonke, Karen Steindorf, Martijn M. Stuiver, Dennis R. Taaffe, Lene Thorsen, Miranda J. Velthuis, Joachim Wiskemann, Ilse Mesters, Cornelia M. Ulrich, Jonna K. van Vulpen, Jose A. E. Custers, Laurien M. Buffart

**Affiliations:** 1 Department of Medical BioSciences, Radboud University Medical Center, Nijmegen, The Netherlands; 2 Division of Psychosocial Research and Epidemiology, Netherlands Cancer Institute, Amsterdam, The Netherlands; 3 Netherlands Cancer Institute/Antoni Van Leeuwenhoek Hospital, Amsterdam, The Netherlands; 4 Institute of Psychiatric and Psychosomatic Psychotherapy, Central Institute of Mental Health, Mannheim, Germany; 5 Heidelberg University, Heidelberg, Germany; 6 Faculty of Health, University of Antwerp, Antwerp, Belgium; 7 Faculty of Kinesiology, Sport, and Recreation, University of Alberta, Edmonton, Canada; 8 Centre for Lifestyle Medicine and Behaviour (CLiMB), The School of Sport, Exercise and Health Sciences, Loughborough University, Loughborough, UK; 9 Exercise Medicine Research Institute, Edith Cowan University, Joondalup, WA Australia; 10 Department of Psychology, University of Groningen, Groningen, Netherlands; 11 University of Twente, Enschede, The Netherlands; 12 School of Public Health, Institute of Health and Biomedical Innovation, Queensland University of Technology, Kelvin Grove, Mackay, QLD Australia; 13 Julius Center for Health Sciences and Primary Care, University Medical Center Utrecht, Utrecht University, Utrecht, The Netherlands; 14 Yale School of Public Health, New Haven, CT USA; 15 Department of Hematology, Cancer Center Amsterdam and LYMMCARE, Amsterdam University Medical Centers, Amsterdam, The Netherlands; 16 Department of Medical Psychology, Amsterdam UMC, University of Amsterdam, Amsterdam, The Netherlands; 17 Robertson Centre for Biostatistics, School of Health and Wellbeing, University of Glasgow, Glasgow, UK; 18 Department of Public and Occupational Health, Amsterdam University Medical Centers, Location VU University, Amsterdam, The Netherlands; 19 Faculty of Health and Behavioural Sciences, School of Human Movement and Nutrition Sciences, University of Queensland, Brisbane, Australia; 20 Department of Human Biology, Faculty of Health Sciences, University of Cape Town, Cape Town, South Africa; 21 School of Public Health, Physiotherapy and Sports Science, UCD, University College Dublin, Dublin, Ireland; 22 Physical Activity for Health Research Center, University of Edinburgh, Edinburgh, UK; 23 School of Human Movement and Nutrition Sciences, The University of Queensland, Brisbane, Australia; 24 Department of Rehabilitation Medicine, UMC Location University of Amsterdam, Meibergdreef 9, Amsterdam, The Netherlands; 25 Amsterdam Movement Sciences, Rehabilitation & Development, Amsterdam, The Netherlands; 26 Division of Physical Activity, Prevention and Cancer, German Cancer Research Center (DKFZ) and National Center for Tumor Diseases (NCT) Heidelberg, a partnership between DKFZ and University Medical Center, Heidelberg, Germany; 27 University of Pittsburgh, Pittsburgh, PA USA; 28 Competence Center for Sports- and Exercise Medicine (Athleticum) and Institute for Medical Psychology, University Medical Center Hamburg-Eppendorf, Hamburg, Germany; 29 National Advisory Unit On Late Effects After Cancer Treatment, Department of Oncology, Division of Cancer Medicine, Oslo University Hospital, Oslo, Norway; 30 Department of Clinical Service, Division of Cancer Medicine, Oslo University Hospital, Oslo, Norway; 31 Netherlands Comprehensive Cancer Organisation (IKNL), Utrecht, The Netherlands; 32 Department of Medical Oncology, Heidelberg University Clinic and National Center for Tumor Diseases (NCT) Heidelberg, a Partnership Between German Cancer Research Center and University Medical Center, Heidelberg, Germany; 33 Department of Epidemiology, Maastricht University, Maastricht, The Netherlands; 34 Huntsman Cancer Institute and Department of Population Health Sciences, University of Utah, Utah, Salt Lake City, USA; 35 Department of Radiation Oncology, University Medical Center Utrecht, Utrecht, The Netherlands; 36 Department of Medical Psychology, Radboud University Medical Center, Nijmegen, The Netherlands

**Keywords:** Distress, Anxiety, Depression, Exercise, Cancer survivors, Moderators

## Abstract

**Purpose:**

This study aimed to investigate whether socio-demographic, clinical, and intervention-related variables moderate the effects of exercise on depression and anxiety symptoms in cancer survivors.

**Methods:**

Data from 26 RCTs in the POLARIS database were analyzed using a one-step individual participant data (IPD) meta-analytic approach with linear mixed models to assess exercise effects on depression and anxiety symptoms (z-scores). Interaction terms were added to these models to explore moderators. Results are presented as betas (corresponding to Cohen’s *d* effect size).

**Results:**

Albeit statistically significant, exercise demonstrated negligible effects on symptoms of depression (*β* = − 0.11; 95% CI = − 0.16; − 0.06) and anxiety (*β* = − 0.07; 95% CI = − 0.12; − 0.02) compared to controls. The effects of exercise interventions on depressive symptoms were larger for patients who were not living with a partner (*β* = − 0.23; 95% CI = − 0.35; − 0.11), had a low/medium education level (*β* = − 0.14; 95% CI = − 0.21; − 0.07), and who had moderate-to-severe symptoms of depression at baseline (*β* = − 0.30; 95% CI = − 0.43; − 0.16). Patients with moderate-to-severe symptoms of depression at baseline combined with those not living with a partner or a low/medium education level yielded the largest effect size through exercise (*β* = − 0.61; 95% CI = − 0.89; − 0.33 and *β* = − 0.37; 95% CI = − 0.57; − 0.17, respectively). Effects on anxiety symptoms were larger for patients with moderate-to-severe symptoms of anxiety at baseline (*β* = − 0.17; 95% CI = − 0.32; − 0.01) compared to those with no-to-mild symptoms. Sex, age, cancer type, BMI, and intervention-related variables did not moderate the exercise effects.

**Conclusion:**

The findings of this study highlight the heterogeneous response to exercise interventions across various patient subgroups. Patients with moderate-to-severe anxiety or depression, those with a low/medium education, and those not living together with a partner may particularly benefit.

**Supplementary Information:**

The online version contains supplementary material available at 10.1007/s00520-025-09646-9.

## Introduction

The rising incidence [1], coupled with advancements in treatment, has led to a growing population of cancer survivors facing burdensome symptoms of cancer and/or side effects of treatment [2]. Following fatigue and pain, symptoms of depression and anxiety emerge as the most prevalent health issues, affecting up to one in five cancer survivors [3]. Symptoms of depression, which are feeling very sad, hopeless, or empty, and anxiety, which is worrying a lot or feeling scared about the future, can significantly impact treatment compliance, well-being, and even serve as predictors for cancer recurrence and mortality [4]. Hence, managing depressive and anxiety symptoms in cancer survivors is of paramount importance.

Exercise is a proven and safe non-pharmacological approach to alleviate symptoms of depression and anxiety during and after cancer treatment [5–7]. The American College of Sports Medicine (ACSM) provides comprehensive international guidelines for cancer survivors for depression and anxiety [8], recommending moderate-intensity aerobic exercise for 30 to 60 minutes three times weekly during treatment. However, it remains unclear how sociodemographic, clinical, and intervention-related variables relate to the effect of exercise on anxiety and depression.

Previous meta-analysis suggests that the effects of exercise on depression and anxiety may be moderated by sociodemographic characteristics such as age, sex, marital status, and education [9], and clinical variables like body mass index (BMI), cancer type [10], and baseline severity of depressive and anxiety symptoms [11]. Intervention-related variables, including exercise type, intensity, duration, and timing (during vs. after treatments) may also play a role. Specific subgroups, including women, individuals under 50 years, those with low socioeconomic status (SES), and those not living together with a partner, report a higher prevalence of depression and anxiety [12–14]. Studies have shown that exercise-induced reductions in depressive symptoms are greater in, or even limited to, patients with high baseline symptoms of distress [15, 16]. ACSM emphasizes the need to investigate such differences, noting that much of the research to date has focused on early-stage cancers and breast cancer survivors, with less attention given to advanced-stage cancers and other cancer types[8]. Understanding these moderating factors could help tailor exercise interventions to maximize their mental health benefits.

The preferred method for exploring moderators is an Individual Participant Data (IPD) meta-analysis. An IPD meta-analysis combines several RCTs by merging and synchronizing IPD, enabling testing of interactions at the participant level, which minimizes the risk of ecological bias [17]. This study, utilizing pooled IPD available through the POLARIS database, explored sociodemographic, clinical, and intervention-related moderators of the exercise effect on symptoms of depression and anxiety in cancer survivors. The aim was to determine for whom and under what conditions exercise interventions are most effective in reducing symptoms of depression and anxiety.

## Methods

### Design

Data from the Predicting OptimaL Cancer RehabIlitation and Supportive care (POLARIS) database [18] were used. This database was primarily set up to evaluate the effect of exercise interventions on the quality of life of adult cancer survivors compared to usual care, waitlist, or an attention control group. It initially comprised 34 RCTs with exercise interventions during or following treatment. Since then, more RCTs have been included in the POLARIS database, identified via literature searches or personal communication, up to 41 RCTs (December 2023). All studies obtained approval from their respective local ethics committees. POLARIS is registered in the Prospective Register of Systemic Reviews (PROSPERO) under reference number CRD 42013003805. A comprehensive description of the study design, search strategy, risk of bias assessment, and procedures has been published previously [18]. A risk of bias assessment for trials newly included in the POLARIS database can be found in Supplementary Table 3. In the present study, RCTs were selected from the POLARIS database based on the assessment of depressive and/or anxiety symptoms. Due to the scarcity of patients with metastatic cancer, patients with metastatic disease were excluded (*n* = 115), resulting in 26 eligible RCTs.

### Outcome variables

The validated questionnaires used in the 26 RCTs are presented in Table 1. To assess symptoms of depression, ten studies [19–29] used the Hospital Anxiety and Depression Scale–depression subscale (HADS-D) [30], eight [31–38] the Centre for Epidemiologic Studies-Depression Scales (CES-D) [39], four [40–43] the Brief Symptom Inventory-18-depression subscale (BSI-18-D) [44], two [45, 46] the Beck Depression Inventory-II (BDI-II) [47], one [48] the Beck Depression Inventory for Primary Care (BDI-PC) [49], and one [50] the Greene Climacteric Scale (GCS) [51]. To assess symptoms of anxiety, ten studies [19–29] used the Hospital Anxiety and Depression Scale–anxiety subscale (HADS-A), five [31–35] the State-Trait Anxiety Inventory (STAI) [52], four [40–43] the Brief Symptom Inventory-18-anxiety subscale (BSI-18-A), one [50] the Greene Climacteric Scale–anxiety subscale (GCS-A), and one [48] the Symptoms checklist–anxiety subscale (SCL-90) [53]. If multiple instruments were used to assess depressive and/or anxiety symptoms within one study, we used the outcome-specific and/or most frequently used questionnaires (Supplementary Table 1). To allow for pooling of the different questionnaires, z-scores were calculated per questionnaire by subtracting its mean score at baseline from the individual values and dividing the result by the mean standard deviation (SD) of the questionnaire at baseline.

**Table 1:** Study characteristics of the included RCTs

Author	Depression questionnaire	Anxiety questionnaire	*N*	Age mean (SD)	Cancer type	Exercise (FITT)	Timing	Mode	Duration (weeks)	Adherence	Control
Mutrie (2007) [46]	BDI-II	-	201	51.6 (9.5)	Breast	F: 2 supervised (+ 1 unsupervised) I: low-moderate T: RE + AE T: 45 min	During CT and/or RT	S	12	Not reported	UC
Daley (2007) [45]	BDI-II	-	108	51.1 (8.6)	Breast	F: 3 ×/week I: moderate-vigorous T: AE T: 50 min	Post	S	8	77% of patients attended ≥ 70% of the exercise sessions	AC vs UC
Goedendorp (2010) [48]	BDI-PC	SCL-90 (A)	144	57.2 (10.5)	Mixed	F: towards 5 × pw I: moderate T: AE T: towards 60 min	During	U	Mean: 31.7	97% of patients attended both counselling sessions	UC
Cormie (2015) [40]	BSI-18 (D)	BSI-18 (A)	64	67.9 (7.1)	Prostate	F: 2 ×/week, I: moderate-vigorous T: RE + AE T: 60 min	During ADT	S	12	Mean number of completed exercise sessions: 23.1 out of 24 offered	UC
Taaffe (2019) [43]	BSI-18 (D)	BSI-18 (A)	104	68.3 (10.2)	Prostate	F: 3 ×/week I: moderate-vigorous T: AE + RE + impact T: 60 min	During/Post ADT	S	26	Mean attended exercise sessions: 79% of the prescribed sessions	UC
Newton (2009) [41]; Taaffe (2017) [43]	BSI-18 (D)	BSI-18 (A)	154	69.0 (9.0)	Prostate	F: 2 ×/week I: moderate-vigorous, T: RE + AE vs RE + impact T: 60 min	During ADT	S	26	Attended supervised exercise sessions: AE + RE: 69% RE + Impact: 65%	WL
Galvao (2014) [42] *RADAR-exercise*	BSI-18 (D)	BSI-18 (A)	100	71.7 (6.4)	Prostate	F:2 ×/week I: moderate-vigorous T: RE + AE T: 60 min	Post ADT	S	26	Mean number of completed exercise sessions: 40.0 ± 11.9 of the 52 exercise sessions (77% attendance)	UC
Schmidt (2015) [37] *BEATE*	CES-D	-	88	52.5 (10.0)	Breast	F: 2 ×/week I: moderate-vigorous T: RE T: 60 min	During CT	S	12	Median attended exercise sessions: 17 out of 24 prescribed sessions (71% attendance)	AC
Steindorf (2014) [38] *BEST*	CES-D	-	141	56.3 (8.9)	Breast	F: 2 ×/week I: moderate-vigorous T: RE T: 60 min	During RT	S	12	Median attended exercise sessions: 19 of 24 offered	AC
Courneya (2003) [34] *CANHOPE*	CES-D	STAI-YI	93	60.3 (10.4)	Colorectal	F: 3–5 ×/week I: moderate T: AE T: 20–30 min	During or post	U	16	75.8% of patients performed on average > 60 min of moderate/strenuous exercise per week	WL
Courneya (2009) [35] *HELP*	CES-D	SF SAI	122	53.2 (14.8)	Haemato- logical	F: 3 ×/week I: moderate-vigorous T: AE T: 15–45 min	During or post	S	12	77.8% of supervised exercise sessions attended	UC
Cadmus (2009) [31] *IMPACT*	CES-D	STAI-YI	50	54.2 (9.6)	Breast	F: aim 5 ×/week I: moderate T: AE T: 30 min	During	U	26	64% of patients met the goal of 150 min of exercise per week	UC
Courneya (2007) [33] *START*	CES-D	SF SAI	242	49.2 (9.3)	Breast	F: 3 ×/week I: moderate-vigorous T: AE vs RE T: 15–45 min	During CT	S	Median: 17	Attendance to supervised exercise sessions: AE: 72.0% RE: 68.2%	UC
Ohira (2006) [36] *WTBS*	CES-D	-	86	52.7 (8.3)	Breast	F: 2 ×/week I:? T: RE T: 60 min	Post	S	26 (13 supervised)	Not reported	WL
Irwin (2009) [32] *YES*	CES-D	STAI-YI	75	55.8 (8.7)	Breast	F: 3 supervised (+ 2 unsupervised) I: moderate T: AE T: 15–30 min	Post	S	26	73% of patients completed at least 80% of the exercise goal of 150 min per week	UC
Hayes (2013) [50] *Exercise for Health*	GCS (D)	GCS (A)	194	52.4 (8.5)	Breast	F: aim: ⩾ 4 ×/week I: moderate T: RE + AE T: 20–45 min	During and/or post	U	35	Attendance to prescribed sessions: FtF: 88% Tel: 81%	UC
Wiskemann (2011) [28]	HADS (D)	HADS (A)	80	48.4 (14.4)	Haemato- logical	F: 5 ×/week I: moderate-vigorous T: RE + AE T: AE: 20–40 min	Pre-during- post	S	Median 16.4	Mean adherence: 87%	AC
Mehnert (2011) [22]	HADS (D)	HADS (A)	58	51.9 (8.5)	Breast	F: 2 ×/week I: moderate T: AE + gymnastics + movement + games + relaxation T: 90 min	Post	S	10	Not reported	WL
Duijts (2012) [19] *EVA*	HADS (D)	HADS (A)	207	47.8 (5.8)	Breast	F: 5 × per 2 weeks I: vigorous T: AE T: 45–60 min	Post	U	12	Mean proportion of patients that were compliant to exercise (i.e. 24 exercise sessions with a metabolic equivalent of 6): 64%	WL
Persoon (2010) [23] *EXIST*	HADS (D)	HADS (A)	109	52.4 (11.2)	Haemato- logical	F: 2 ×/week I: moderate-vigorous T: RE + AE T: 60 min	Post SCT	S	18	Patients attended 86% of the prescribed exercise sessions	UC
Thorsen (2005) [24]	HADS (D)	HADS (A)	139	39.4 (8.3)	Mixed	F: 2 ×/week or more I: moderate-vigorous T: RE + AE T: 30 min or more	Post	U	14	97% adhered to the minimum of 2 exercise sessions per week	UC
Korstjens (2008) [20] *OncoRev*	HADS (D)	HADS (A)	133	50.6 (10.2)	Mixed	F: 2 ×/week I: AE M/V, RE L/M T: RE + AE T: 12 min	Post	S	12	83.5% of 24 exercise sessions completed	WL
Van Waart (2015) [27] *PACES*	HADS (D)	HADS (A)	253	51.4 (9.5)	Breast and colon	F: 2 ×/week I: moderate-vigorous T: RE + AE T: 60 min (supervised) F: towards 5 ×/week I: moderate T: AET T: aim 30 min (unsupervised)	During CT	U/S	Mean: 15.9	On Track: 71% of the prescribed supervised sessions attended. 48% of patients followed daily activity recommendations ≥ 75% of the time Oncomove: 55% of patients followed daily activity recommendations ≥ 75% of the time	UC
Travier (2015) [25]; van Vulpen (2015) [26] *PACT*	HADS (D)	HADS (A)	237	50.7 (8.8)	Breast and colon	F: 2 ×/week I: moderate-vigorous T: RE + AE T: 60 min	During CT	S	18	83% of the prescribed supervised exercise sessions attended	UC
van Vulpen (2021) [29] *PERFECT*	HADS (D)	HADS (A)	120	64 (8)	Oesophageal	F:2 ×/week (+ 5 ×/week u.) I: moderate-vigorous T: RE + AE T: 60 min	Post surgery	S	12	96% of the prescribed exercise sessions attended	UC
Kampshoff (2015) [21] *REACT*	HADS (D)	HADS (A)	277	53.5 (11.0)	Mixed	F: 2 ×/week I: moderate vs vigorous T: RE + AE, T: 60 min	Post	S	12	Proportion of patients attending ≥ 80% of supervised sessions HI: 74% LMI: 70%	WL

*FITT*, frequency, intensity, type and time of exercise; *AE*, aerobic; *RE*, resistance training; *CT*, chemotherapy; *ADT*, androgen deprivation therapy; *RT*, radiotherapy; *U*, unsupervised exercise; *S*, supervised exercise; *UC*, usual care; *WL*, wait-list; *AC*, attention control; *HADS*, Hospital Anxiety and Depression Scale; *CES-D*, Centre for Epidemiologic Studies-Depression Scales; *BSI-18*, Brief Symptom Inventory-18; *BDI-II*, Beck Depression Inventory-II; *BDI-PC*, Beck Depression Inventory for Primary Care; *GCS*, Greene Climacteric Scale; *STAI*, State-Trait Anxiety Inventory; *SCL-90*, Symptoms checklist–anxiety subscale; *D*, depression subscale; *A*, anxiety subscale; *FtF*, face-to-face; *Tel*, over-the-telephone; *CBT*, cognitive behavioral therapy; *PE*, physical exercise, *HI*, high intensity; *LMI*, low-to-moderate intensity

### Moderators

Sociodemographic variables included age (both continuous and categorized into young adult (≤39), middle-aged adult (39–65), and older adults (≥65) [54], sex (male/female), marital status (living without a partner/living with a partner), and education (low/medium-high). For clarification, the two marital status groups are referred to as living with or without a partner in this paper.

Clinical moderators included cancer type (breast, male genitourinary, gastrointestinal, hematological, or mixed), BMI (continuous), and categorized moderate-to-severe depressive or anxiety symptoms at baseline through a questionnaire (yes/no). Determination of moderate-to-severe symptoms of depression or anxiety at baseline was based on the following available cut-off scores per questionnaire: CES-D, ≥16 [55]; BSI-18, ≥50 [56, 57]; STAI, ≥41 [58]; HADS, ≥8 [59]; BDI-PC, ≥4 [49]; and BDI, ≥10 [60]. SCL90 (*n* = 1) and GCS (*n* = 1) were not included in this moderator analysis as no predefined cut-offs were available. Higher scores on all scales represent more depressive or anxiety symptoms.

Intervention-related variables included intervention timing (during/post primary cancer treatment), mode of delivery (supervised/unsupervised), type of exercise (with aerobic component or without aerobic component), intensity (low-moderate and moderate/moderate-to-vigorous intensity), and volume, (dichotomized into <150/≥150 minutes per week, following ACSM guidelines [8]). All moderators were chosen a priori with clear rationale and aligned with previous studies to allow for meaningful comparison.

### Statistical analysis

The described procedures involved two separate analyses: one examining the outcome of depressive symptoms and the other examining anxiety symptoms. We performed a complete-case one-step IPD meta-analytic approach, adhering to the intention-to-treat principle, using all data available from the studies without imputing missing data. A linear mixed model analysis was used with a random intercept on the study level to account for the clustering of patients within studies. The independent variables were allocation (intervention/control) and continuous baseline scores of depressive or anxiety symptoms (z-scores). The dependent variables were continuous post-intervention scores of depression or anxiety (z-scores). Results are presented as between-group differences in z-scores, corresponding to a Cohen’s *d* effect size. Effect sizes were interpreted as small (0.2–0.5), moderate (0.5–0.8), and large (≥ 0.8) [61], with values below 0.2 considered negligible. To mitigate ecological bias, individual values of the sociodemographic and clinical moderators were centered around their mean study value to separate within-trial interaction from between-trial interaction [62]. However, intervention-related moderators were exempt from centering, as they exhibited no within-study variation.

Potential sociodemographic and clinical moderators were tested individually by adding each moderator and its interaction term with group allocation as independent variables to separate models. Stratified analyses were performed for each moderator subgroup if models exhibited significant improvements after adding the interaction term based on the likelihood ratio test (*p* < 0.05), signifying the relevance of the moderator. Considering the multiple exercise arms in some studies, potential intervention-related moderators were explored using dummy variables instead of interaction terms. Due to the presence of multiple statistically significant moderators, we performed a post-hoc sensitivity stratified analysis for marital status and educational level in those with moderate-to-severe symptoms of depression. All analyses were executed using R version 4.1.1.

## Results

### Study characteristics

A total of 26 RCTs evaluated the effect of exercise intervention on depressive symptoms, of which 21 also evaluated anxiety symptoms. These 26 RCTs represented 3530 patients in total, of whom 3167 had complete depressive symptom data (3% missing at baseline and 10% after the exercise intervention) and 2565 had complete anxiety symptom data (3% missing at baseline and 12% after the exercise intervention). Overall, 1953 patients were assigned to the intervention group and 1577 to the control group. The majority of studies included patients with breast cancer (*n* = 11), followed by mixed types of cancer (*n* = 5), and prostate cancer (*n* = 4). The most common control group type was usual care (*n* = 15), and the most frequently employed questionnaire was the HADS (*n* = 10). Most exercise interventions were delivered during cancer treatment (*n* = 15) and were predominantly or completely supervised (*n* = 20). The duration of the intervention ranged from 8 to 35 weeks, as summarized in Table 1.

### Patient and intervention characteristics

The 3530 included patients had a mean age of 54.2 (SD, 11.7) and a mean BMI of 26.9 (SD, 4.8). Notably, 72.3% of the patients were female, most patients lived with a partner (67.0%), and almost half had a low/medium education level (49.7%). At baseline, 26.0% presented moderate-to-severe symptoms of depression, while 19.5% presented moderate-to-severe symptoms of anxiety (Table 2). The median scores for symptoms of depression and anxiety before and after the exercise intervention are outlined in Table 3. The interventions predominantly featured low-volume exercise training (<150 minutes per week; 65.3%) and exercise training with an aerobic component (84.9%; Table 2).

**Table 2:** Sociodemographic, clinical, and intervention-related variables of individual participants

	Intervention (*n* = 1953)	Control (*n* = 1577)
**Sociodemographic**		
Sex, *n* (%)		
Female	1412 (72.3%)	1139 (72.2%)
Male	541 (27.7%)	438 (27.8%)
Age, mean years (SD)	54.3 (11.9)	54.1 (11.5)
Age categories years, *n* (%)		
≤ 39	194 (9.9%)	161 (10.2%)
39–65	1353 (69.3%)	1130 (71.7%)
≥ 65	404 (20.7%)	280 (17.8%)
N/A	2 (0.1%)	6 (0.4%)
Marital status, *n* (%)		
Living without a partner	356 (18.2%)	312 (19.8%)
Living with a partner	1317 (67.4%)	1047 (66.4%)
N/A	280 (14.3%)	218 (13.8%)
Education level, *n* (%)		
Low/medium	970 (49.7%)	786 (49.8%)
High	754 (38.6%)	538 (34.1%)
N/A	229 (11.7%)	253 (16.0%)
**Clinical**		
Cancer type *n* (%)		
Breast	1236 (63.3%)	996 (63.2%)
(Male) Genitourinary	280 (14.3%)	214 (13.6%)
Gastrointestinal	200 (10.2%)	139 (8.8%)
Hematological	199 (9.9%)	195 (12.4%)
Mixed	38 (1.9%)	33 (2.1%)
BMI, mean (SD) kg/m^2^	26.7 (4.7)	27.0 (5.0)
BMI categories, *n* (%)		
Underweight	11 (0.6%)	16 (1.0%)
Normal	638 (32.7%)	495 (31.4%)
Overweight	588 (30.1%)	481 (30.5%)
Obese	352 (18.0%)	303 (19.2%)
N/A	364 (18.6%)	282 (17.9%)
Moderate-to-severe symptoms of depression at baseline *n* (%)		
Yes	465 (23.8%)	452 (28.7%)
No	1322 (67.7%)	1021 (64.7%)
N/A	166 (8.5%)	104 (6.6%)
Moderate-to-severe symptoms of anxiety at baseline *n* (%)		
Yes	385 (19.7%)	303 (19.2%)
No	1052 (53.9%)	777 (49.3%)
N/A	516 (26.4%)	497 (31.5%)
**Intervention-related variables**		
Timing		
During treatment	1150 (58.9%)	-
Post treatment	760 (38.9%)	
N/A	43 (2.2%)	
Intensity		
Low and low-moderate	627 (32.1%)	-
Moderate-to-vigorous	1211 (62.0%)	
N/A	115 (5.9%)	
Volume*		
150 min per week	1275 (65.3%)	-
≥ 150 min per week	531 (27.2%)	
N/A	147 (7.5%)	
Mode of delivery		
Supervised	1419 (72.7%)	-
Non-supervised	534 (27.3%)	
Type of exercise		
Aerobic component	1659 (84.9%)	-
No aerobic component	294 (15.1%)	

*BMI*, body mass index; *N/A*, not available

^*^Cut-off value based on ACSM guidelines[8]

**Table 3:** Descriptive information for each specific questionnaire used to assess depression and anxiety before and after the exercise intervention

Depression	Before median (IQR)	After median (IQR)	Anxiety	Before median (IQR)	After median (IQR)
HADS-D Range: 0–21	3 (1–6)	2 (1–6)	HADS-A Range: 0–21	5 (3–8)	4 (2–7)
CES-D Range: 0–60	12 (6–20)	12 (4–19)	STAI Range: 20–80	34 (26–43)	30 (24–40)
BSI-18-D Range: 0–18	0 (0–2)	0 (0–2)	SAI Range: 10–80	31 (21–46)	27 (20–39)
BDI-II Range: 0–63	11 (6–16)	7 (3–13)	BSI-18-A Range: 0–18	1 (0–2)	0 (0–2)
BDI-PC Range: 0–21	0 (0–1)	0 (0–1)	SCL-90 Range: 7–35	12 (10–15)	11 (10–13)
GCS-D Range: 0–15	3 (1–5)	2 (1–5)	GCS-A Range: 0–18	3 (1–5)	3 (1–4)

Higher scores on all scales represent more depressive or anxiety symptoms. Abbreviations: *HADS*, Hospital Anxiety and Depression Scale; *CES-D*, Centre for Epidemiologic Studies-Depression Scales; *BSI-18*, Brief Symptom Inventory-18; *BDI-II*, Beck Depression Inventory-II; *BDI-PC*,1 Beck Depression Inventory for Primary Care; *GCS*, Greene Climacteric Scale; *STAI*, State-Trait Anxiety Inventory; *SCL-90*, Symptoms checklist–anxiety subscale; *D*, depression subscale; *A*, anxiety subscale

### Overall exercise effects on symptoms of depression and anxiety

Overall, albeit statistically significant, exercise demonstrated negligible effects on depressive (*β* = −0.11 [95% CI = −0.16; −0.06]) and anxiety symptoms (*β* = −0.07 [95% CI = −0.12; −0.02]) immediately after the intervention when compared to the control group (Table 4).

**Table 4:** Sociodemographic and clinical moderators of the exercise effects on symptoms of depression and anxiety

	Depression			Anxiety		
Overall effect	β –0.11; 95% CI –0.16; –0.06			β –0.07; 95% CI –0.12; –0.02		
	Interaction β (95%CI)	*P*-value LRT	Stratified analysis β (95%CI)^a^	Interaction β (95%CI)	*P*-value LRT	Stratified analysis β (95%CI)^a^
Sociodemographic variables						
Sex	–0.07 (–0.24; 0.11)	0.44	*Not performed*	–0.06 (–0.23; 0.11)	0.49	*Not performed*
Age (continuous)	0.003 (–0.002; 0.008)	0.29	*Not performed*	0.004 (–0.001; 0.009)	0.13	*Not performed*
Age (category)						
18–39	Reference	0.12		Reference	0.08	
39–66	0.17 (0.01; 0.34)			0.20 (0.03; 0.38)		
≥ 66	0.16 (–0.05; 0.37)			0.16 (–0.06; 0.38)		
Marital Status					0.83	*Not performed*
Living without a partner	Reference	0.01	–0.23 (–0.35; –0.11)*	Reference		
Living with a partner	0.16 (0.04; 0.29)		–0.06 (–0.13; –0.02)*	0.01 (–0.12; 0.14)		
Educational status		0.01			0.13	*Not performed*
Low/medium	Reference		–0.14 (–0.21; –0.07)*	Reference		
High	0.13 (0.03; 0.24)		–0.03 (–0.11; 0.04)	0.09 (–0.02; 0.20)		
Clinical variables						
BMI	0.006 (–0.006; 0.017)	0.34	*Not performed*	–0.011 (–0.024; 0.002)	0.11	*Not performed*
Cancer type		0.96	*Not performed*		0.81	*Not performed*
Breast	Reference			Reference		
(male) Genitourinary	–0.06 (–0.44; 0.32)			0.08 (–0.29; 0.44)		
Gastrointestinal	–0.01 (–0.28; 0.27)			0.15 (–0.11; 0.41)		
Haematological	0.06 (–0.31; 0.42)			0.10 (–0.25; 0.45)		
Mixed	–0.11 (–0.49; 0.26)			–0.00 (–0.36; 0.36)		
Cancer type		0.73	*Not performed*		0.34	*Not performed*
Breast	Reference			Reference		
Mixed (other)	–0.03 (–0.23;0.16)			0.09 (–0.10;0.28)		
Moderate or severe symptoms at baseline						
No	Reference	< 0.01	–0.06 (–0.11; –0.01)*	Reference	0.02	–0.053 (–0.111; 0.004)
Yes	–0.23 (–0.38; –0.09)		–0.30 (–0.43; –0.16)*	–0.17 (–0.32; –0.03)		–0.17 (–0.32; –0.01)*

^a^Analysis only performed when *p*-value from likelihood ratio test (LRT) was statistically significant (< 0.05)

*Indicates statistically significant effect

### Sociodemographic moderators

The exercise effect on depressive symptoms was moderated by marital status and education level. Patients living without a partner showed a small statistically significant reduction in depressive symptoms (*β* = −0.23 [95% CI = −0.35; −0.11]), while those living with a partner experienced a negligible, though statistically significant, effect (*β* = −0.06 [95% CI = −0.13; −0.02]). Similarly, patients with a low or medium education level exhibited a negligible yet significant reduction in depressive symptoms (*β* = −0.14 [95% CI = −0.21; −0.07]), whereas those with a high education level showed no statistically significant effect (*β* = −0.03 [95% CI = −0.11; 0.04]). Neither sex nor age moderated the impact of x`exercise on depressive symptoms (Table 4). Additionally, none of the sociodemographic variables were found to moderate the effect of exercise on anxiety symptoms (Table 4).

### Clinical moderators

The exercise effects on symptoms of depression and anxiety were moderated by moderate-to-severe symptoms of depression and anxiety, respectively, at baseline. Small statistically significant effects were observed in patients with moderate-to-severe symptoms of depression at baseline (*β* = −0.30 [95% CI = −0.43; −0.16]) compared to negligible effects in those with no or mild symptoms of depression at baseline (*β* = −0.06 [95% CI = −0.11; −0.01]). The same was observed for symptoms of anxiety (moderate-to-severe symptoms of anxiety: *β* = −0.17 [95% CI = −0.32; −0.01], no or mild symptoms of anxiety: *β* = −0.05 [95% CI = −0.11; 0.00]). Cancer type and BMI did not statistically significantly moderate the exercise effects on symptoms of depression and anxiety (Table 4).

### Intervention-related moderators

None of the intervention-related variables exhibited statistically significant differences from one another, although variations in effect sizes were noted (Table 5).

**Table 5:** Intervention-related moderators of the exercise effects on symptoms of depression and anxiety

	Depression	Anxiety
	*β* (95% CI)	*β* (95% CI)
Overall effect	− 0.11 (− 0.16; − 0.06)	− 0.07 (− 0.12; − 0.02)
**Intervention-related variables**		
Timing	Reference − 0.09 (− 0.16; − 0.03)* − 0.14 (− 0.21; − 0.06)*	Reference − 0.05 (− 0.12; 0.01) − 0.12 (− 0.19; − 0.05)*
Control group		
During treatment		
Post treatment		
Volume	Reference − 0.09 (− 0.15; − 0.04)* − 0.13 (− 0.21; − 0.05)*	Reference − 0.05 (− 0.11; 0.01) − 0.11 (− 0.19; − 0.03)*
Control group		
< 150 min/week		
≥ 150 min/week		
Mode of delivery	Reference − 0.06 (− 0.15; 0.03) − 0.12 (− 0.18; − 0.07)*	Reference − 0.06 (− 0.15; 0.02) − 0.08 (− 0.14; − 0.02)*
Control group		
Unsupervised		
Supervised		
Intensity	Reference − 0.14 (− 0.22; − 0.06)* − 0.10 (− 0.15; − 0.04)*	Reference − 0.11 (− 0.19; − 0.03)* − 0.06 (− 0.11; 0.00)
Control group		
Low–low-moderate		
Moderate-to-vigorous		
Type of exercise	Reference − 0.12 (− 0.17; − 0.07)* − 0.04 (− 0.14; 0.07)	Reference − 0.07 (− 0.12; − 0.02)* − 0.07 (− 0.22; 0.07)
Control group		
Aerobic component group		
No aerobic component		

The asterisk * indicates statistically significant effect

### Post hoc stratified analysis

Post hoc stratified analysis for marital status and education in those with moderate-to-severe symptoms of depression showed moderate effects on symptoms of depression in patients living without a partner (*β* = −0.61 [95% CI = −0.89; −0.33]) and small effects in patients with low to medium educational levels (*β* = −0.37 [95% CI = −0.57; −0.17]) (Supplementary Table 2). Conversely, negligible effects were found in patients living with a partner (*β* = −0.08 [95% CI = −0.24; 0.07) and patients with high educational levels (*β* = −0.06 [95% CI = −0.25; 0.12]), respectively.

## Discussion

This study explored the overall effects of exercise training on symptoms of depression and anxiety, as well as potential moderators of these effects, using pooled IPD of multiple trials. The analysis included 26 RCTs involving 3530 patients with cancer. We found negligible overall effects of exercise on symptoms of depression and anxiety, with significant heterogeneity observed for both effects. These findings align with and support conclusions drawn from prior meta-analyses relying on aggregated data, particularly focusing on depressive symptoms, which reported effect sizes ranging from negligible to small (−0.13 to −0.38) [5, 63–67].

In our IPD analysis, patients with a low to medium education level, those living without a partner, and experiencing moderate-to-severe symptoms of depression at baseline showed greater reductions in depressive symptoms as a results of exercise. The largest benefits from participation in exercise were seen in individuals who had moderate-to-severe depression combined with either a low to medium educational level or patients living without a partner; these effects sizes ranged from small to moderate, respectively. Similarly for anxiety symptoms, patients with moderate-to-severe symptoms of anxiety at baseline showed greater improvements following an exercise intervention. Sex, age, cancer type, BMI, and intervention-related variables did not moderate the exercise effects on symptoms of depression and anxiety.

### Sociodemographic variables

Our finding that patients living without a partner and with a low to medium educational level demonstrated greater benefits from exercise in reducing symptoms of depression aligns with explorative analyses from prior single RCTs [33, 68, 69]. These subgroup differences may be explained by the tendency of patients living with a partner or with higher levels of education to report fewer symptoms of depression and anxiety [13, 70]. This pattern was also reflected in our data, where 30% of patients living without a partner had moderate-to-severe depression, compared to 24% of those living with a partner. Additionally, individuals with low to medium educational levels tend to have lower baseline physical activity, providing more potential for improvement through exercise training [71]. Age and sex did not play a moderating role in the exercise effect on symptoms of depression or anxiety, consistent with previous IPD meta-analyses on various outcomes such as sleep, fatigue, and health-related quality of life [9, 72, 73].

### Clinical variables

Patients with moderate-to-severe symptoms of depression or anxiety at baseline demonstrated a greater reduction in depressive and anxiety symptoms, respectively. This finding is consistent with a previous RCT, which reported that cancer patients with moderate-to-severe depression or anxiety experienced nearly a 50% reduction, compared to a 6% increase in the control group [74]. In contrast, patients with low baseline levels of depression and anxiety had limited potential for further improvement, a phenomenon known as the floor effect. Consequently, because many patients had low baseline symptoms of depression and anxiety, the overall effect sizes may appear small. Importantly, the primary outcome of included studies was not specifically anxiety or depression, and the studies typically did not focus exclusively on recruiting patients with high baseline distress symptoms [8, 74]. This broad inclusion likely diluted the overall effect size, as the benefits of exercise were much more pronounced in patients with moderate-to-severe symptoms compared to those with mild symptoms.

### Intervention-related variables

Findings from previous meta-analyses show that supervised or partially supervised interventions, those not conducted at home, focusing on aerobic exercise, and lasting at least 30 minutes yield larger effects on depressive symptoms [65, 66]. However, in our study, none of the intervention-related variables were found to moderate the effects of exercise on symptoms of depression and anxiety. This highlights that engaging in any form of exercise contributes to the overall trend of positive outcomes for depressive and anxiety symptoms, regardless of specific intervention characteristics.

### Mechanisms involved between exercise and symptoms of depression and anxiety

Exercise and symptoms of depression or anxiety are suggested to be linked through various mechanisms, spanning from biological to psychosocial pathways [75, 76]. Among these, inflammation-related factors may play a significant role [77], with studies linking inflammatory markers to depression and anxiety [78, 79]. Exercise contributes to an anti-inflammatory environment [80]. In addition to the inflammation pathway, exercise influences neurochemicals such as endorphins and serotonin [76]. Endorphins, known for their mood-boosting properties, are released during exercise, contributing to feelings of well-being and alleviating symptoms of depression and anxiety [81]. Other hormones such as testosterone, which increase in the circulation during and after exercise, also have suppressive effects on depression and anxiety [82, 83]. On the behavioral front, engaging in exercise programs and mastering new movement skills fosters a sense of mastery [84], while perceived improvements in strength and flexibility elevate physical self-esteem and consequently global self-esteem [85]. Moreover, the social support element of supervised exercise programs can provide additional emotional and psychological benefits, further aiding the reduction of depressive and anxiety symptoms.

### Clinical implication

The observed effect sizes of exercise on symptoms of depression and anxiety range from negligible to moderate, highlighting the heterogeneous nature of the response. Beyond alleviating symptoms of depression and anxiety, exercise provides additional benefits, such as reducing fatigue, improving physical symptoms, and restoring body self-confidence. These multifaceted advantages underscore the value of exercise as an intervention, emphasizing the importance of integrating exercise into comprehensive strategies for cancer survivors.

### Strengths, limitations and future research

The availability of a large set of IPD in our study offered a unique opportunity to investigate if the effect of exercise interventions on symptoms of depression and anxiety differed significantly across subgroups of patients with cancer. Single randomized controlled trials (RCTs) and conventional meta-analyses are often underpowered and limited to the use of aggregate data, respectively [87]. We performed uniform analytical procedures across the included RCTs, comprising a substantial total sample size. This approach allowed for the examination of sociodemographic and clinical variables at the patient level, thereby mitigating the risk of ecological bias. Ecological bias pertains to associations at the study level that may not accurately mirror associations at the patient level due to the confounding of study characteristics [87]. Nevertheless, the results should be interpreted considering some limitations. First, we used IPD available through the POLARIS database, rather than performing an original IPD meta-analysis including new literature searches and assessments of study representativeness and publication bias. Therefore, these biases cannot be ruled out. Second, the categorization of variables may result in loss of information or the grouping of non-homogeneous entities. However, we prioritized categories reflective of clinical practice. Third, the data analysis was limited to exploring single interactions, potentially overlooking multiple interactions. In addition, the data analysis was limited to immediate post-intervention measurements. Thus, the persistence of the immediate exercise effect beyond the intervention period remains unassessed. Additionally, intervention-related variables differed only between, not within, studies making these results prone to ecological bias. To gain a deeper understanding of which aspects of exercise interventions influence the effect on symptoms of depression and anxiety, future research should compare these characteristics within studies while keeping other factors constant.

## Conclusions

The findings of this study underscore the heterogeneous response to exercise interventions across various patient subgroups, highlighting the influence of factors like marital status, education level and moderate-to-severe symptoms of depression and anxiety at baseline on the exercise effect on depressive symptoms. While exercise should be encouraged for most patients with cancer, those with moderate-to-severe anxiety or depression, low to medium education levels and those not living with a partner could particularly benefit. Understanding these nuances is crucial for guiding exercise recommendations and setting realistic expectations for benefits. By incorporating these factors into discussions between exercise professionals and participants, interventions can be better tailored to individual needs, ultimately enhancing the effectiveness of exercise programs in alleviating symptoms of depression and anxiety.

## Supplementary Information

Below is the link to the electronic supplementary material.

Supplementary file1 (DOCX 39 KB)

## Data Availability

No datasets were generated or analysed during the current study.
