# Supplementary material for: Exercise effects on symptoms of depression and anxiety vary by patient, clinical, and intervention characteristics in cancer survivors: Results from pooled analyses of individual participant data of 26 RCTs
Source: Support Care Cancer. 2025 Jul 1;33(7):647. doi: 10.1007/s00520-025-09646-9 (PMC12213934; doi:10.1007/s00520-025-09646-9)
Supplement: Supplementary file 1 — Supplementary file1 (DOCX 39 KB) [file 520_2025_9646_MOESM1_ESM.docx]

**Supplemental Tables**

# Supplementary Table 1: Order for selecting questionnaires when multiple instruments were used in study

| HADS | Hospital Anxiety and Depression Scale |
| --- | --- |
| CES-D | Centre for Epidemiologic Studies-Depression Scales |
| STAI | State-Trait Anxiety Inventory |
| BSI-18 | Brief Symptom Inventory-18 |
| BDI-II | Beck Depression Inventory-II |
| BDI-PC | Beck Depression Inventory for Primary Care |
| GCS | Greene Climacteric Scale |

Supplementary Table 2: Results of post-hoc sensitivity stratified analyses by marital status and educational level in patients with moderate-to-severe symptoms of depression

|  | Depression  β (95%CI) |
| --- | --- |
| Moderate to severe symptoms at baseline AND living without a partner | -0.61 (-0.89;-0.33)* |
| Moderate to severe symptoms at baseline AND living with a partner | -0.08 (-0.24; 0.07) |
| Moderate to severe symptoms at baseline AND low/medium education | -0.37 (-0.57;-0.17)* |
| Moderate to severe symptoms at baseline AND high education | -0.06 (-0.25; 0.12) |

Supplementary Table 3: Risk of bias assessment of two trials assessing distress that were newly added to the POLARIS database.

|  | RSG | AC | IO | IR | Adh | Con |
| --- | --- | --- | --- | --- | --- | --- |
| Taaffe et al., 2019 | + | + | + | + | - | ? |
| Van Vulpen et al., 2021 | + | + | + | + | + | - |
| Quality assessment: + = low risk of bias; - = high risk of bias; ? = unclear quality; RSG = random sequence generation; AC = allocation concealment; IO = incomplete outcome; IR = incomplete reporting; Adh = adherence; Con = contamination. | | | | | | |
